# A critique of International Classification of Diseases 11th Revision criteria for the mood disorders

**DOI:** 10.1177/00048674251356403

**Published:** 2025-07-09

**Authors:** Gordon Parker

**Affiliations:** Discipline of Psychiatry and Mental Health, School of Clinical Medicine, University of New South Wales, Sydney, NSW, Australia

**Keywords:** Depressive disorders, bipolar disorders, ICD-11, CDDR manual

## Abstract

In 2024, WHO published the Clinical Descriptions and Diagnostic Requirements for International Classification of Diseases 11th Revision Mental, Behavioural and Neurodevelopmental Disorders manual. Stated objectives were to determine valid and reliable diagnoses and defined so as to differentiate conditions both from other conditions and from normative states. This paper provides a critique of the criteria sets derived for both the depressive and the bipolar disorders. It is argued that failure to specify a minimum number of criteria for certain conditions compromises valid intrinsic and differential definitions, while additional reasons leading to the quantified low reliability of dysthymia are noted. Several idiosyncratic definitions and rulings are noted, while numerous boundary issues (differentiating disorder categories and clinical depression from normative depression) are noted. The use and application of ‘postcoordination’ codes (in addition to ‘specifiers’) appears enigmatic and potentially unserviceable.

## Introduction

Following a major revision, the World Health Organization (WHO) published the Clinical Descriptions and Diagnostic Requirements for International Classification of Diseases 11th Revision (ICD-11) Mental, Behavioural and Neurodevelopmental Disorders (CDDR) manual in March 2024 (World Health Organization, 2024). As overviewed recently by Kestel and Reed (2024), the CDDR criteria were designed to support health professionals in ‘accurately diagnosing mental disorders’. Following the appointment of 16 expert working groups, proposed diagnostic criteria were tested in field studies involving 19,000 mental health and primary care professionals from 165 countries, while reliability studies evaluated levels of diagnostic agreement between two clinicians attending the same clinical interview.

The CDDR manual notes (p. 4) that the working groups were charged with reviewing the literature to ‘recommend changes to advance the validity of ICD-11’, and (p. 1) that ‘the reliability, clinical utility and global applicability of the CDDR have been confirmed through a comprehensive programme of developmental and evaluative field studies’. Furthermore, the manual notes (p. 2) that derived criteria place an emphasis on ‘how to differentiate disorders from non-pathological expressions of human experience and from other disorders including medical conditions’, and thus weight addressing ‘boundary’ issues.

ICD-11 coding is highly complex. The manual’s first ‘character’ effectively indicates chapter positioning, the second and third indicate the diagnostic class or grouping (e.g. ‘depressive disorders’), the fourth ‘indicates the specific disorder within the class’ (e.g. single depressive disorder), the fifth character ‘indicates subtypes or specifiers’ (e.g. single episode, mild), the sixth captures status (e.g. currently symptomatic or in remission), while there are also ‘postcordination’ codes which go ‘beyond just capturing clinical information within the confines of a single diagnostic code by allowing additional codes to be linked to the initial diagnostic code for the purpose of indicating additional clinically significant features’ (p. 31).

This review will focus on how ICD-11’s CCDR manual classifies the depressive and bipolar mood disorders in relation to its stated objectives (i.e. reliability, validity and boundary issues) and offer several challenges.

## CCDR criteria for the depressive disorders

While the CCDR manual lists multiple depressive conditions, this critique will prioritize consideration of the key depressive category – a ‘depressive episode’. Its 10 criteria comprise two in an ‘affective’ cluster, four in a ‘cognitive-behavioral’ cluster and four in a ‘neurovegetative’ cluster. Five must have been present concurrently for at least most of the day and nearly every day over a 2-week period, and at least one must be from the ‘affective cluster’. The CCDR requires the episode to be impairing or, if ‘functioning is maintained, it is only through significant additional effort’ (p. 213). Judging impairment (by clinician or patient) is therefore subjective and with attendant risks.

While well recognized symptoms of clinical depression are listed, an individual could meet diagnostic criteria at the symptom level if they (a) felt down or sad, (b) had reduced sexual desire, (c) were markedly indecisive, (d) were sleeping excessively and (e) had significant weight gain, a not particularly substantive set. In arguing that the CCDR criteria set for a ‘depressive episode’ provide a ‘low’ diagnostic bar I further note that criteria for a mild depressive episode include (p. 216) that ‘none of the symptoms of a depressive episode should be present to an intense degree’, that the individual is ‘usually distressed by the symptoms’ (allowing that they may not be distressed at all) and that the individual has ‘some difficulty’ in functioning. As with *Diagnostic and Statistical Manual of Mental Disorders, Fifth Edition*, (*DSM*-5) criteria for a major depressive disorder there is a risk of pathologizing human misery and over-diagnosing ‘normal depression’ as clinical depression – a ‘boundary’ issue.

There is also the converse risk of those with severe clinical depression not meeting CCDR diagnostic criteria for a depressive episode. For example, an individual who developed a suicidal and incapacitating depressive state 4 days prior to assessment would be ineligible for such a diagnosis as the mandatory duration criterion of 2 weeks had not been met. As a colleague noted in relation to another topic, a hurricane is not defined by its duration. Common sense argues for an appropriate rider to the duration criterion.

‘Severity, psychotic symptoms and remission specifiers’ for both ‘single episode depressive disorder’ and ‘recurrent depressive disorder’ allow for ‘mild’, ‘moderate’ and ‘severe’ expressions. The latter two are differentiated from the ‘mild’ expressions and from each other by symptom numbers being greater (but no minimum numbers are mandated), more marked and/or more intense, and by there being ‘considerable difficulty’ and ‘serious difficulty’ respectively (as against ‘some difficulty’) in functioning across various domains – again involving subjective judgements. Allowing ‘some difficulty’ as a boundary is at variance with the base diagnosis requirement of ‘significant impairment’.

While the presence of ‘psychotic symptoms’ is a ‘Severity, psychotic symptoms and remission’ specifier for a ‘single episode depressive disorder’ and a ‘recurrent depressive disorder’, melancholia is not included in either list but is later listed in a second list of five ‘symptomatic and course presentation’ specifiers for single episode and recurrent depressive disorders, being given alternate ‘postcordination’ status (6A80.3, pp. 46 and 47). Six symptoms are listed for melancholia (p. 249): essentially anhedonia, non-reactivity, terminal insomnia, diurnal variation, marked appetite/weight loss and marked psychomotor disturbance) but no minimum symptom number is required, merely the presence of ‘several’. As all are also included in the list of ‘essential’ symptoms for defining the base condition, this allows that the symptom profile for some patients meeting criteria for a single or recurrent depressive episode *with* melancholia may not differ from patients experiencing a single or recurrent depressive episode *without* melancholia and so dissallowing any ‘boundary’.

Dysthymic disorder’s definition requires a persistent depressed mood (i.e. lasting 2 years or longer) occurring for most of the day and for more days than not. The manual lists a number of exemplar features but imposes no minimum symptom number. An individual with a persistent depressed mood and experiencing low self-worth would meet dysthymia criteria but the criteria set could also simply capture a personality style. In essence, the boundary between ‘caseness’ and ‘non-caseness’ lacks precision. Symptom-free periods of up to 2 months are allowed – inviting the question as to how such expressions of dysthymia might be differentiated from ‘recurrent depressive disorder’. The manual states (p. 252) that such differentiation is based on the number of symptoms (but no number is specified for dysthymia) and the ‘course of the disorder’ (but, as noted, the initially suggested 2-year period is subsequently modified to allow quite extensive symptom-free periods). As noted, the development process included multiple field trials evaluating inter-rater reliability. Of all conditions so evaluated, the lowest level of agreement was for dysthymic disorder – the kappa coefficient being 0.45 (p. 7). Such a result is likely to reflect in part the definitional ambiguities.

## A critique of CCDR criteria for the bipolar disorders

The manual defines a bipolar type I disorder by a history of at least one manic or mixed episode (and provides a set of ‘essential features’) and a bipolar type II episode by at least one hypomanic episode and at least one depressive episode (also with a set of ‘essential features’), and with the latter diagnosis excluded if there is a history of manic or mixed episodes.

For a manic episode, two features are required – an ‘extreme mood state. . . . characterized by “euphoria, irritability or expansiveness” and, second, increased activity or a subjective experience of increased energy’ (p. 217). For a hypomanic state the same two features are required (p. 222) as for a manic state (i.e. persistent elevation of mood or increased irritability and increased activity or the subjective experience of increased energy). In addition, the manual lists seven other ‘essential’ symptoms (effectively describing pressured speech, flight of ideas, grandiosity, decreased need for sleep, distractibility, impulsive reckless behaviour and increased libido or goal-directed activity) for both mania (p. 217) and hypomania (p. 222), thus providing no symptom differentiation between mania and hypomania, and allowing that only ‘several’ (p. 217) should be present rather than specifying a minimum number – both issues evidencing ‘boundary’ limitations. Specifiers allow that a bipolar I manic episode may or may not have psychotic symptoms.

While a period of 1 week is imposed for a manic episode, it is stated that if manic or mixed episodes have been previously experienced. then ‘a duration of 1 week is not required’ (p. 226) for diagnostic purposes. For a hypomanic episode, symptoms should be present for ‘at least several days’ (p. 222), which could, of course, exceed a week. The lack of any clear differential indicates that duration is not viewed as a differentiation criterion for the bipolar conditions. While a CCDR criterion for mania is that it ‘results in significant impairment’ (p. 218), the relevant CCDR criterion for hypomania states (p. 223) that the mood disturbance is ‘not sufficiently severe to cause marked impairment’, another subjective judgement that risks blurring the boundary between the two conditions operationally.

‘Rapid cycling’ is defined (p. 235) as involving at least four ‘mood episodes’ over the preceding 12 months. It is noted (p. 235), however, that if ‘depressive and manic episodes alternate very rapidly (i.e. from day to day or within the same day) a mixed episode should be diagnosed rather than rapid cycling’. As ‘rapid cycling’ is usually defined on the basis of rapid changes in polar states, and mixed states by the contemporaneous presence of both hypo/manic and depressive symptoms, the CCDR terminology is idiosyncratic. A diagnosis of a bipolar II disorder is disallowed if the individual has not experienced clinical depressive episodes or if mixed states have been experienced.

There are 18 specifiers for bipolar I disorder and 12 for bipolar II disorder (with both allowing psychotic symptoms but neither providing any melancholia listing). However, a second set of ‘symptomatic and course presentation specifiers’ include melancholia as a ‘postcordination’ coding for both bipolar conditions and with the six listed (pp. 62, 63) symptoms being the same as for the depressive disorders but without any minimum number imposed. An additional clinical nuance (p. 237) is that a mixed episode in a patient ‘initially diagnosed with bipolar II disorder’ requires a diagnostic change to a bipolar type I disorder, whereas *DSM*-5 allows (p. 136) the presence of mixed features in those with a bipolar II disorder.

The CCDR manual lists a Cyclothymic Disorder, defined (p. 240) as ‘Mood instability over an extended period of time . . . characterized by numerous hypomanic and depressive episodes’ and present ‘for more days than not’ (p. 240). At first pass, such a presentation appears comparable to a bipolar II state in having both hypomanic and depressive phases. The manual states (p. 240) that ‘Hypomanic periods may or may not have been sufficiently severe or prolonged to meet the diagnostic requirement for a hypomanic episode’. In allowing ‘hypomanic’ episodes, and that both severity and duration ‘may’ be akin to a hypomanic episode, where lies the hypomania boundary differentiating cyclothymia from a bipolar II disorder?

Furthermore, it is noted (p. 241) that ‘Individuals with cyclothymic disorder do not typically exhibit psychotic symptoms’, a statement that therefore allows their presence.

## Discussion

We expect a diagnostic psychiatric manual to capture material psychiatric conditions validly – in the sense of providing criteria that capture their integral features, define them reliably and differentiate them from other class conditions and from normative states.

The CCDR model for the depressive conditions invites a number of questions and challenges. Both ‘Single episode depressive disorder’ and ‘Recurrent depressive disorder’ may be mild, moderate or severe – but their boundaries are minimally defined, subjectively weighted and thus imprecise. While five of ten listed symptoms are required, not all are substantive. For ‘mild’ depressive episodes, no symptom is required at an ‘intense degree’, sufferers are ‘usually distressed’ (which allows that they may not be distressed) and experience ‘some difficulty’ in functioning. Such low-level descriptors risk pathologizing ‘normal’ depressive mood states – a boundary issue.

While *DSM*-5 lists a melancholia specifier (for ‘major depression’) its status in the CCDR manual is unclear. It is not included in the list of 17 specifiers but is listed (for both the depressive and bipolar disorders) as one of seven ‘postcoordination’ codes (6A80.3). Seeking clarification as to how the latter status differs from a specifier, I accessed a paper by Mabon et al. (2022), a key source as the third author chaired the ICD-11 Revision Steering Group and was employed by WHO. In that paper the authors defined a ‘postcoordination code’ as a specific ICD-11 term combining or linking ‘two or more codes into a cluster that describes a clinical concept’. In illustrating how such codes are formulated and applied, they offered an example of a patient experiencing a closed transverse intertrochanteric fracture of the right hip – a diagnosis that would generate a stem code. That individual would then receive a stem code for the fracture, an extension code for its laterality, an extension code for the fracture subtype, an extension code for it being an open or closed fracture, a stem code for it being an unintentional fall from less than one metre, an extension code for the injury being caused by a loose floor covering, and an extension code for the place of occurrence and so generating a postcoordination coding of NC72.30&XK9K&XJV7&XJ44E/PA60&XE3WK&XE66.

The complexity of manual coding is illustrated by it providing ‘precordinated’, ‘post coordinated’ as well as ‘extension’ codes (p. 32) and their actual application. For example, the manual illustrates (p. 32) how an individual experiencing a schizophrenic episode with varying symptoms might receive the following code: ‘6A20.00/6A25.0&XSOT/6A25.1&YS25/6A25.2&YS8H/6A25.4&XS5W/6A25&XS25’. Although it is noted there that ‘It is not expected that such complex codes will be used routinely by individual clinicians recording diagnoses by hand’, a stated innovation of ICD-11 (p. 30) was ‘its ability to capture much more clinical information associated with a particular diagnosis than was possible with ICD-10’. Such extension aspirations clearly raise concerns about design overreach (and unservability) but are intrinsically compromised if the diagnostic criterion building blocks are problematic, as argued in this paper.

The manual has seven ‘postcoordination’ codes (capturing prominent anxiety symptoms, panic attacks, persistence, seasonal episode, rapid cycling, as well as melancholia) for both single-episode and recurrent depressive disorders, as well as for both the bipolar and bipolar II disorders. Such latter exemplars do not begin to match the exemplar provided by Mabon et al. (2022) for a surgical condition and all might more simply have been listed as ‘specifiers’. Melancholia’s definition remains unclear as only ‘several’ of a set of symptoms are required and, as all are listed as symptoms for a depressive episode, depressive episodes ‘with’ and ‘without’ melancholia risk non-differentiation, again challenging the extent to which the manual addresses boundaries.

Other boundary concerns are evident in relation to dysthymia. The initial definition indicates that such a condition would need to be present for 2 years, but we are later informed that symptom-free periods are allowed while no minimum number of symptoms is required. These definitional components generate another boundary challenge – in essence, how might so-defined dysthymia and recurrent depressive disorder differ if euthymic intervals are allowed for dysthymia?

The lack of any required number of symptoms for dysthymia and melancholia is likely to have reflected an issue detailed in the manual’s Introduction and where it is stated that professionals involved in international multilingual studies ‘preferred more flexible guidance to allow for cultural variation and clinical judgment compared to a strict criteria-based approach, and were receptive to a system incorporating a dimensional approach’ (pp. 5–6). Thus, criteria numbers are variably imposed or not and providing a very variegated model.

Turning to the bipolar disorders, a number of points will be made. First, the listed CCDR defining symptoms for mania and hypomania (and thus for bipolar I and II status) are identical, and only ‘several’ are required to be present, again challenging the manual’s objective to define boundaries between clinical conditions. Psychotic features are allowed for both bipolar I and bipolar II states, but not mandated for either (when their presence might be a categorical marker of a manic state). Differentiation by minimal duration is minimal, as episodes of hypomania are allowed to last ‘at least several days’ – and which could of course be a week – the minimum required for a manic episode.

While those with a bipolar II defined condition must have had depressive episodes, this is not a requirement for a bipolar I diagnosis, allowing a unipolar bipolar I condition but disallowing without explanation a unipolar bipolar II condition. While the latter pattern is rare in clinical practice, it is observed and with the absence of depressive episodes perhaps artefactually reflecting individuals not having been followed for long-enough periods.

The manual disallows those with a bipolar II state having mixed states (their presence alone changing their diagnosis to a bipolar I state), a ruling at variance with a review of salient studies by Goodwin and Jamison (2007) and who noted (p. 81) that ‘hypomanic behavior and/or symptoms intruding into depressive episodes . . . have been described since Kraepelin’s 1899 and Weygant’s 1899 contributions’.

The CCDR criteria for mania require ‘significant impairment’ while hypomania should not show ‘marked impairment’. Cleavage between the two descriptors is problematic, while there is a more salient issue in play. Both Jamison et al. (1980) and Judd et al. (2005) established that functioning can actually improve in mania and hypomania, and its frequency is underlined by our earlier study (Parker et al., 2022) where we reported that 76% of those with a bipolar I condition and 86% of those with a bipolar II condition reported improved functioning during hypo/manic states. This reality led us to suggest (p. 4) that this domain might be better handled by stating that such mood states are associated with a ‘clear change in functioning’ – which allows impairment but does not mandate it. Moving away from a criteria-based system to provide ‘more flexible guidance’ (as noted in the manual – p. 6) demands precision in terminology, and the example just provided illustrates how descriptors benefit from nuanced expression.

While there are six symptom criteria listed for melancholia in those with unipolar and bipolar disorders, no minimum number is required, again challenging the manual’s objective of providing boundaries. Repositioning very rapid cycling (albeit not operationalized) as a mixed state challenges long-standing definitions – of rapid cycling involving frequent switching from one pole to another and a mixed state requiring the coterminous presence of depressive and hypo/manic symptoms. Where lies the argument for changing such long-standing definitions?

The manual’s employment of the descriptor ‘several’ (e.g. in relation to number of symptoms that might be required for melancholic, manic and hypomanic episodes as well as for the number of days in determining any hypomanic episode) clouds any objective of generating reliable diagnostic criteria. Statistical techniques (such as machine learning) can operationally determine optimal cut-off symptom numbers for diagnostic categories while, for example, the clinical observation that some individuals may experience hypomanic and even manic episodes for less than a day allows clinical observation to potentially shape reliable criteria. Further clouding in the manual is evidenced by the use of terms such as ‘usually’ and there being ‘some difficulty’. In allowing that those with a depressive episode of sufficient severity as to warrant a formal psychiatric diagnosis are ‘usually distressed’ the criterion effectively allows that some may instead be emotionally sanguine while, as impairment is allowed to be expressed at a level of generating only ‘some difficulty’, caseness boundaries risk distinct blurring.

No psychiatric manual is without its faults or limitations in addressing such objectives. *DSM* is not uncommonly referred to as ‘the Bible’, which implies a religious weighting to capturing and preserving ‘eternal truths’ while science is distinguished by allowing that ‘truths’ should be open to challenge and potential modification. Respect for the latter should hopefully allow critiques such the one provided here, so that current limitations to criteria and operational guidelines can be addressed in future editions of the ICD manual.

## Conclusions

Focussing only on the ICD-11’s manual consideration of the mood disorders, a number of the stated objectives can be challenged and with many evidencing ‘boundary’ concerns. These include questioning the validity of some diagnostic categories (especially when no minimum number of criteria may be imposed), some idiosyncratic definitions, some definitions compromising reliability (as quantified for dysthymia), the risk of pathologizing normative depressive mood states, the failure to impose a minimum number of symptoms required for certain diagnoses and the unclear status of postcoordination codes as against ‘specifiers’.
